# Non-cytopathic bovine viral diarrhea virus (BVDV) inhibits innate immune responses via induction of mitophagy

**DOI:** 10.1186/s13567-024-01284-z

**Published:** 2024-03-05

**Authors:** Zhijun Li, Ying Zhang, Bao Zhao, Qinghong Xue, Chunjiang Wang, Siyu Wan, Jingyu Wang, Xiwen Chen, Xuefeng Qi

**Affiliations:** 1 College of Veterinary Medicine, Northwest A&F University, Yangling, Shaanxi China; 2 Key Laboratory of Ruminant Disease Prevention and Control (West), Ministry of Agriculture and Rural Affairs, Xi’an, China; 3 Shaanxi Animal Disease Control Center, Xi’an, China; 4 China Institute of Veterinary Drug Control, Beijing, China; 5 Hebei Veyong Pharmaceutical Co., Ltd, Shijiazhuang, China; 6 Animal Disease Prevention and Control & Healthy Breeding Engineering Technology Research Center, Mianyang Normal University, Mianyang, Sichuan China

**Keywords:** BVDV, cGAS, innate immunity, MAVS, mitophagy, PINK1-Parkin, persistent infection

## Abstract

Bovine viral diarrhea virus (BVDV) belongs to the genus *Pestivirus* within the family *Flaviviridae*. Mitophagy plays important roles in virus-host interactions. Here, we provide evidence that non-cytopathic (NCP) BVDV shifts the balance of mitochondrial dynamics toward fission and induces mitophagy to inhibit innate immune responses. Mechanistically, NCP BVDV triggers the translocation of dynamin-related protein (Drp1) to mitochondria and stimulates its phosphorylation at Ser616, leading to mitochondrial fission. In parallel, NCP BVDV-induced complete mitophagy via Parkin-dependent pathway contributes to eliminating damaged mitochondria to inhibit MAVS- and mtDNA-cGAS-mediated innate immunity responses, mtROS-mediated inflammatory responses and apoptosis initiation. Importantly, we demonstrate that the LIR motif of E^RNS^ is essential for mitophagy induction. In conclusion, this study is the first to show that NCP BVDV-induced mitophagy plays a central role in promoting cell survival and inhibiting innate immune responses in vitro.

## Introduction

Bovine viral diarrhea virus (BVDV) is an economically important pathogen of cattle that can infect a wide range of domestic and wild species including sheep, goats, deer, camelids, pigs, and wildlife animals [1, 2]. A remarkable feature of BVDV is the existence of two biotypes, cytopathic (CP) and non-cytopathic (NCP), as defined by its effect on cultured cells [3]. Although viral biotype does not correlate with virulence in vivo, the establishment of persistent infection is restricted to the NCP strain [4]. In general, establishment of persistent infection requires successful production of progeny and overriding of the innate immune responses of the host [5]. It has been suggested that innate immune activation and apoptosis induction possibly attribute to distinct pathogenesis between CP and NCP BVDV [6]. In contrast to the induction of apoptosis by CP BVDV, the cells infected with NCP BVDV do not undergo apoptosis [3, 6]. Furthermore, accumulated in vivo and in vitro evidence has implicated that NCP BVDV does not induce the synthesis of interferon (IFN) in its host cells, suggesting that the evasion of the innate immune system may be crucial for the establishment of persistent infection [3].

Mitochondria form a dynamic network that constantly undergoes rearrangement and turnover [7]. It has been demonstrated that mitochondria fulfill multiple cellular functions, such as energy production, maintenance of calcium homeostasis, reactive oxygen species (ROS) generation and apoptosis initiation [8, 9]. In addition, mitochondria also actively participate in innate immunity in order to restrict viral infection [10]. Thus, the rapid modulation of mitochondrial dynamics occurs in response to physiological stress, apoptotic stimuli, metabolic demands and viral infection [11]. Mitophagy, a selective autophagy, mainly regulates mitochondrial quality control by specifically targeting and eliminating damaged and dysfunctional mitochondria via cross-talk with the autophagic machinery [12]. It has been demonstrated that there are two types of mitophagy regulatory pathways, classified as PINK1-PRKN/PARK2 (parkin RBR E3 ubiquitin protein ligase)-mediated mitophagy and receptor-mediated mitophagy [13]. PINK1/Parkin-mediated mitophagy plays a key role in regulating mitochondrial quality control in mammalian cells [14]. Under healthy conditions, PINK1 is imported into the mitochondrial inner membrane (MIM) and degraded by mitochondrial proteases; upon mitochondrial dysfunction or low mitochondrial membrane potential, PINK1 accumulates on the mitochondrial outer membrane (MOM) and recruits Parkin to damaged mitochondria to mediate Parkin-dependent mitophagy for the elimination of damaged mitochondria [14, 15]. Ever-increasing amounts of evidence have demonstrated the involvement of mitophagy in viral pathogenesis, such as hepatitis B virus (HBV), hepatitis C virus (HCV) and classical swine fever virus (CSFV) [16–18].

In this study, we determine the involvement of aberrant mitochondrial dynamics and mitophagy in Madin–Darby bovine kidney (MDBK) cells infected with NCP BVDV. Our data reveal that NCP BVDV shifts the balance of mitochondrial dynamics toward fission and promotes the formation of mitophagosomes via the Parkin-dependent mitophagy pathway. It is interesting to note that NCP BVDV infection effectively degrades mitophagosomes via induction of complete mitophagy. Importantly, NCP BVDV-induced complete mitophagy can effectively inhibit type I IFN expression, inflammatory cytokines expression and apoptosis initiation. To the best of our knowledge, this study is the first to show that NCP BVDV-induced mitophagy may play key roles in inhibiting innate immune responses in vitro.

## Materials and methods

### Cell lines and virus

Madin–Darby bovine kidney (MDBK) cells were provided by the American Type Culture Collection (ATCC; CCL-22). HEK 293T cells were purchased from the China Center for Type Culture Collection (CCTCC, Beijing, China). The cells were cultured in Dulbecco modified Eagle medium (DMEM; Life Technologies Corporation, Gaithersburg, MD, USA) supplemented with 10% fetal bovine serum (FBS) (Gibco, 10270-106), 100 IU/mL penicillin and 100 μg/mL streptomycin (Hyclone, SV30010). Cells were cultured at 37 °C with 5% CO_2_ in a humidified incubator (Thermo Fisher Scientific, Waltham, MA, USA).

The New York 1 strain of BVDV (NY-1, genotype 1b and NCP type) used in this study was obtained from the China Veterinary Culture Collection Centre (CVCC). It belongs to genotype 1b (GenBank accession no. FJ387232) and was propagated in MDBK cells. Infected cells and supernatants were harvested and freeze-thawed three times. The viral titers were determined by IFA assay, cells cultivated in 96-well plates were inoculated with tenfold serial dilutions of the virus and incubated at 37 °C for 5 to 7 days. The viral titers were estimated with the Reed and Muench method [19, 20]. The MOI was confirmed according to the viral titers of the respective cell lines.

### Antibodies and reagents

The following primary antibodies were used: NCP BVDV NS4B antibody was prepared in our laboratory. Anti-MFN2 (Proteintech, 12186-1-AP), anti-VDAC1 (Proteintech, 55259-1-AP), anti-p62 (Proteintech, 18420-1-AP), anti-Flag (Proteintech, 66008-4-Ig), anti-caspase 3 (Proteintech, 19677-1-AP), anti-TBK1 (Ser172) (ImmunoWay, YP1527), anti-DRP1 (Ser616) (Immunoway, YP1318), anti-IRF-3 (Ser396) (ImmunoWay, YP0326), anti-DRP1 (Immunoway, YT1414), anti-PINK1 (Cell Signaling Technology, 6946), anti-HA (Cell Signaling Technology, 3724S), Anti-Parkin (Abcam, ab77924), anti-cGAS (D-9) (Santa Cruz, sc-515777), anti-TBK1 (Santa Cruz, sc-398366), anti-cleaved PARP (Abways, CY5035), Anti-β-Actin (Invitrogen), anti-LC3B (Merck-Sigma-Aldrich, L7543), Anti-GAPDH (Abcom, ab8245).

The following secondary antibodies were used: HRP-conjugated goat anti-mouse IgG (Merck-Sigma-Aldrich, A9917), HRP-conjugated goat anti-rabbit IgG (Merck-Sigma-Aldrich, A0545), fluorescein isothiocyanate (FITC)-conjugated goat anti-rabbit IgG (Merck-Sigma-Aldrich, F9887), fluorescein isothiocyanate (FITC)-conjugated goat anti-mouse IgG (Merck-Sigma-Aldrich, F0257), Alexa Fluor 647 AffiniPure Donkey Anti-Mouse IgG (H+L) (Yeasen Biotechnology, 34113ES60).

Chemicals and reagents: Chloroquine (Merck-Sigma-Aldrich, C6628), Rapamycin (MedChemExpress, HY-10219) and 3-MA (MedChemExpress, HY-19312), carbonyl cyanide 3-chlorophenylhydrazone (CCCP, Merck-Sigma-Aldrich, C2759).

### Virus infection and cell treatment

MDBK cells were seeded on 24-well plates at a density of 1 × 10^5^ cells/well and infected with NCP BVDV at a multiplicity of infection (MOI) of 5 or mock-infected with phosphate-buffered saline (PBS). Then, MDBK cells were maintained in DMEM supplemented with 2% FBS, 100 IU/mL penicillin and 100 µg/mL streptomycin at 37 °C with 5% CO_2_ for the indicated times. Then, cell samples were fixed or collected for immunofluorescence and Western blot assays, respectively.

### RNA isolation and real-time PCR analysis

Total RNA was extracted from cells using Trizol reagent (Invitrogen, Waltham, MA, USA) according to the manufacturer’s instructions. RNA was then reversed using Superscript III (Invitrogen) and random primers (Invitrogen). Real-time quantitative PCR assays were carried out using an ABI 7500 System (Applied Biosystems, Warrington, UK) and Power SYBR green PCR master mix (Applies Biosystems). The sequences of the primers and reaction conditions for bovine cytokines (IFN-β, IL-18 and IL-1β) and housekeeping gene (glyceraldehyde phosphate dehydrogenase [GAPDH]) have been previously described [21, 22]. N^pro^ gene of NCP BVDV was used to detect the viral replication level. The reaction conditions were previously described [23]. The primer sequences of N^pro^ gene used in this study are as follow:

- N^pro^, forward, 5′-ATCCGCAGTCAACGCTAAAA-3′;
- N^pro^, reverse, 5′-GGCCCTGGTTTTAAATAGATTCC-3′.

### RNA interference

Small interfering RNA (siRNA) targeting *Drp1* (target site: CAGCGAGACTGT-GAAGTTA) and *Parkin* (target site: GCAGAGAAGTCGGGATCTACA) were designed and synthesized by Tsingke, Inc. (Beijing, China). siRNA were used for silencing the target genes as previously described [19]. Briefly, MDBK cells were transfected with 50 nM siRNA targeting Drp1 and Parkin by using Turbofect reagent according to the manufacturer’s guidelines (Invitrogen, Carlsbad, CA, USA). Then, MDBK cells were cultured in DMEM medium supplemented with 10% FBS for 24 h, and infected with NCP BVDV (MOI = 5) for 48 h before the cells were harvested for Western blot assays.

### Western blot analysis

Protein homogenates from the cells were extracted as previously described [19]. Briefly, the cells were lysed for 20 min on ice in ice-cold lysis buffer (Roche). The lysates were centrifuged at 12 000 × *g* for 20 min at 4 °C to obtain a clear lysate. The protein content of each sample was determined using the BCA protein assay kit (Thermo Scientific). Then, equal amounts of protein were separated on a 12% SDS–polyacrylamide gel and transferred to polyvinylidene difluoride (PVDF) membranes. Membranes were probed overnight at 4 °C with primary antibodies followed by HRP-conjugated secondary antibodies. The bound antibodies were detected with ECL protocol (Amersham Biosciences, Piscataway, NJ, USA). Signal was visualized using Konica SRX 101A developer (Konica Minolta Medical Imaging, Wayne, NJ, USA) and the Quantity One software (Bio-Rad, Mississauga, ON, Canada) was used for densitometrical analysis. All target proteins and internal loading controls were detected and verified within the same linear range.

### Immunofluorescence assay

Following the indicated treatments, MDBK cells were stained with 200 nM Mito Tracker Red CMXRos (Beyotime, C1035), fixed in 4% paraformaldehyde and treated with 0.1% Triton X-100 for 15 min. Then the cells were incubated with 1% bovine serum albumin (BSA; Merck-Sigma-Aldrich, A7906) and the appropriate primary antibodies for 1 h at 37 °C. Subsequently, the cells were washed and incubated simultaneously with FITC- or Alexa Fluor 647-conjugated secondary antibodies. Finally, the cells were treated with Hoechst 33342 (Merck-Sigma-Aldrich, B2261) solution and analyzed under a confocal microscope (A1R; Nikon, Japan). The mitochondrial mean length and the ratio of co-localization at indicated treatment cells were performed using Image J software Version 1.53t (NIH) as previously described [24].

### Lentivirus and stable cell line construction

Lentiviral production was performed as previously described [25]. Briefly, HEK-293T cells were transfected with empty pLenti-Puro for control or pLenti-Puro containing the HA-Drp1, HA-Parkin and Flag-E^RNS^ genes, together with psPAX2 and pMD2.G (Addgene, Cambridge, MA, USA) by using Turbofect transfection reagent. Viral supernatants were collected at 48 h. MDBK cells containing representative genes were generated by incubation with lentiviral supernatants supplemented with polybrene and puromycin. The protein expression levels were verified by Western blot.

### TUNEL assay

One-step terminal deoxy nucleotide transferase-mediated d-UTP biotin nick end labeling (TUNEL) staining was performed in the present study to determine the apoptosis of mock or NCP BVDV-infected cells using One Step TUNEL Apoptosis Assay Kit (Beyotime, C1090). The staining procedures were performed as previously described [26]. The cells were analysed under a confocal microscope (A1R; Nikon, Japan).

### Flow cytometry analysis

Flow cytometry analysis was performed to detect the intracellular BVDV protein, cells were incubated with  anti-BVDV-NS4B antibody and stained with FITC-conjugated anti-mouse IgG antibody. Then, the cells were analyzed using BD FACS Aria™ III High Speed Cell Sorter (BD Biosciences, San Diego, CA, USA).

Apoptosis analysis was performed using the annexin V-FITC double-staining apoptosis detection kit (Beyotime, C1062S) according to the manufacturer’s instructions. Briefly, MDBK cells were infected with NCP BVDV in the presence or absence of siParkin or siNC, the cells were resuspended in 500 μL of 10× binding buffer, then stained with 5 μL of FITC-labelled annexin V and 10 μL of propidium iodide (PI) for 15 min. Subsequently, fluorescence intensity of all staining samples was measured using BD FACS Aria™ III High Speed Cell Sorter (BD Biosciences, San Diego, CA, USA), followed by analysis with FlowJo software, version 10 (Treestar, San Carlos, CA, USA).

### Autophagic flux measurements

For monitoring the progression from autophagosomes to autolysosomes using an mRFP-GFP-LC3 vector as previously described [27, 28]. MDBK cells were seeded on 24 well plates and infected with plenti-mRFP-GFP-LC3 lentivirus. Then the MDBK cells were infected with NCP BVDV (MOI = 5) for 48 h. The autophagic flux was visualized by laser confocal microscopy (A1R; Nikon, Japan).

### Co-immunoprecipitation assay

HEK293T cells were transfected with HA-E^RNS^ and Flag-LC3-II encoding plasmids for 48 h and incubated on ice with immunoprecipitation lysis buffer (Beyotime, P0013). For each sample, 500 μL of lysate was incubated with the appropriate antibodies and protein A/G plus agarose (Santa Cruz Biotechnology, sc-2003) overnight. The agarose beads were washed four times with 1 mL of lysis buffer containing 1% NP-40 (Beyotime, ST366). The precipitates were detected by Western blot.

### Mitochondria isolation

The mitochondrial fractions were isolated using the cell mitochondria isolation kit (Beyotime, C3601) according to the manufacturer’s instructions. Briefly, MDBK cells were resuspended in mitochondrial lysis buffer and homogenized with a microhomogenizer, then placed in an ice bath for 15 min. Subsequently, the cell homogenate was centrifuged at 600 × *g* for 10 min at 4 °C. The supernatant was collected and centrifuged at 11 000 × *g* for 10 min at 4 °C to isolate the mitochondrial fractions (pellet) by removing the cytoplasmic fractions (supernatant). Then the mitochondrial fractions and cytoplasmic fractions were subjected to Western blot analysis.

### Analysis of mitochondrial DNA expression in the cytosol

Mitochondrial isolation was performed as previously described [29]. Briefly, MDBK cells were harvested at individual time points, then washed with PBS, and DNA isolated using Quick-DNA™ Miniprep Kit (Zymo Research, D3025). Mitochondrial DNA levels were measured by comparing the relative levels of mitochondrial DNA with nuclear DNA by qPCR. The mitochondrial DNA amplicons were determined from two distinct segments of the mitochondrial DNA genes: mt-CO1 and mt-ND6. GAPDH was used as a nuclear amplicon as well as the internal control. Each reaction was carried out in triplicate. Primers used in this study are as follows:

- mt-CO1, forward, 5′-GTAGTTGTAACCGCACACGC-3′; mt-CO1, reverse, 5′-TTGCCTGCTAAGGGAGGGTA-3′.
- mt-ND6, forward, 5′-AAAGCCGCAATCCCTATGGC-3′; mt-ND6, reverse, 5′-AGGGGCATTTGTTACTGGCT-3′.

### Caspase activity detection

Caspase colorimetric assay kit (Beyotime, C1115) was used to detect the activities of caspase 3. MDBK cells were pre-transfected with siParkin or siNC for 24 h prior to viral infection; then cells were infected with NCP BVDV (MOI = 5). At the indicated time points, the cells were treated with lysis buffer, and the protein concentrations were measured using BCA protein assay reagent (Beyotime, P0012). Then, 150 μg lysates of each sample were loaded into microplates and incubated with each caspase substrate at 37 °C for 4 h; after that, the absorbance values of the samples were measured at 405 nm in a microplate spectrophotometer (Infinite 200 PRO Nano Quant, Tecan, Switzerland).

### Statistical analysis

The data are expressed as the means ± standard deviation (SD) of three independent experiments. The significance of the variability between the different treatment groups as calculated with one-way ANOVA, followed by Tukey multiple comparisons test using GraphPad Prism 6.0 software (GraphPad Software Inc., San Diego, CA, USA). *P < 0.05; **P < 0.01; ns: nonsignificant.

## Results

### NCP BVDV infection induces the formation of mitophagosomes

**Figure 1:**
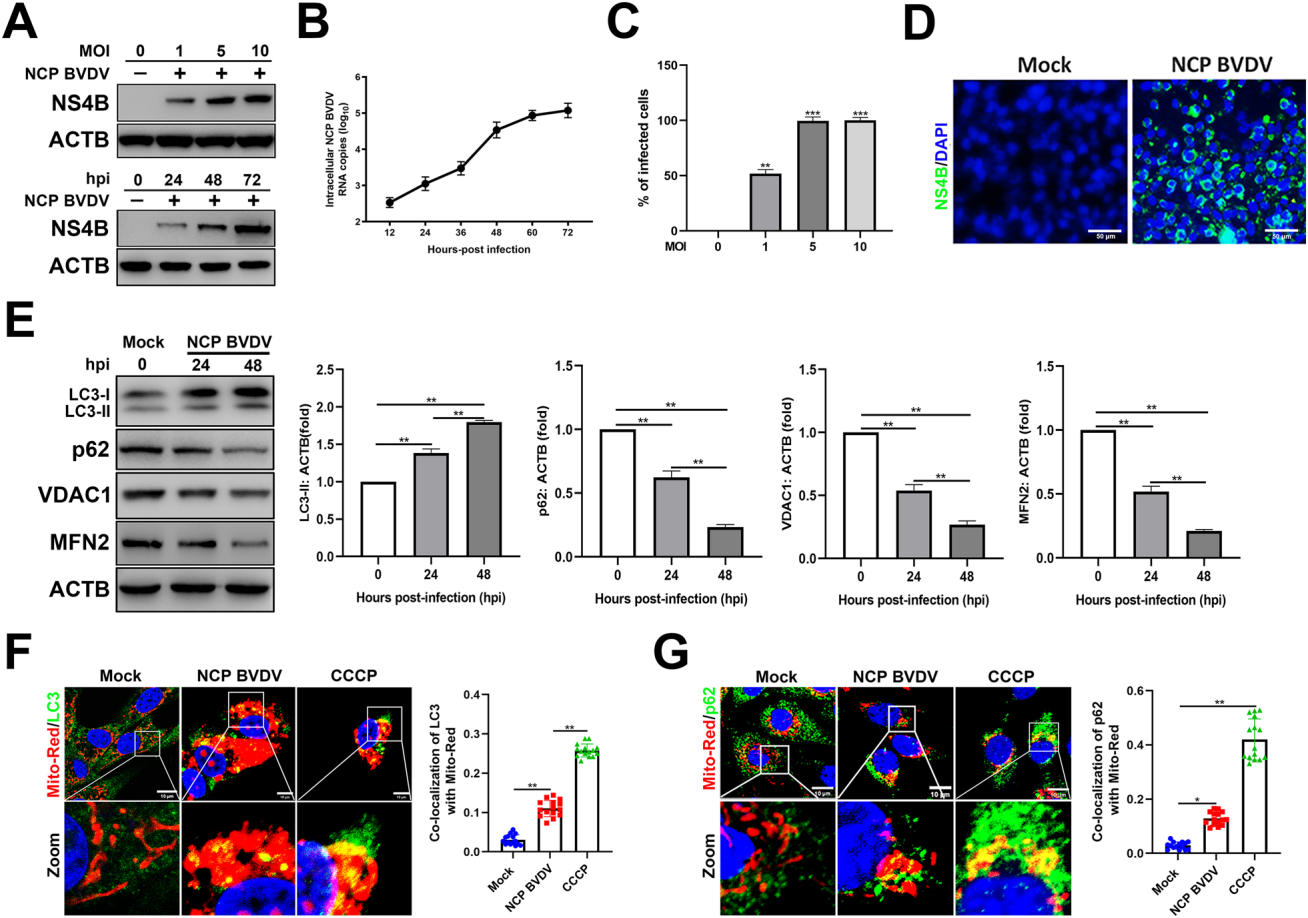
**NCP BVDV infection induces the formation of mitophagosomes.** **A** Western blot analysis of NS4B protein in mock and NCP BVDV-infected (MOI = 1, 5, 10) cells at 48 hpi (upper) or at 0 hpi, 24 hpi, 48 hpi and 72 hpi (MOI = 5) (under). Equal amounts of protein from mock and NCP BVDV-infected cells were separated using SDS-PAGE and transferred to PVDF membranes. The membranes were probed with NS4B antibody. **B** RNA from NCP BVDV-infected (MOI = 5) cells was collected, and BVDV N^pro^ gene was measured by qRT-PCR. **C** NCP BVDV-infected (MOI = 1, 5, 10) cells were bound with BVDV NS4B antibody and then labelled with fluorophore-conjugated antibody or matched isotype controls and analyzed by flow cytometry at 48 hpi. **D** IFA analysis of NS4B expression in cells infected with NCP BVDV (MOI = 5) at 48 hpi (Scale bar = 50 μm). **E** Western blot analysis of LC3-II, p62, VDAC1 and MFN2 proteins in mock and NCP BVDV-infected cells at 24 hpi and 48 hpi. **F**, **G** IFA analysis of the recruitment of LC3-II to damaged mitochondria (**F**) and the recruitment of p62 to damaged mitochondria (**G**) in mock and NCP BVDV-infected (MOI = 5) cells at 48 hpi. (Scale bar = 10 μm). Data are given as means ± standard deviation (SD) from three independent experiments. *P* values were calculated using Student *t* test. An asterisk indicates a comparison with the indicated control. *P < 0.05; **P < 0.01; ***P < 0.001; ns: not significant.

To determine the relationship between BVDV infection and mitochondrial dysfunction, we first determined the virus replication levels in NCP BVDV-infected cells using Western blot and qPCR assays. The kinetics of virus replication indicated that an increased virus replication level of NCP BVDV was detected in a dose-dependent or in a post infection time-dependent manner (Figures 1A, B). Furthermore, flow cytometry analysis showed that the percentage of infected cells following NCP BVDV infection increased up to 100% with an MOI of either 5 or 10 at 48 hpi (Figure 1C). Immunofluorescence analysis also showed that NCP BVDV infection efficiency reached 100% with an MOI of 5 at 48 hpi (Figure 1D).

Previous studies have shown that BVDV infection induces autophagy [30]. Here, we consistently found that the expression of p62, MFN2 and VDAC1 was significantly reduced in NCP BVDV-infected cells compared to that in mock-infected cells. Meanwhile, a significantly increased expression of microtubule associated protein 1 light chain 3 (MAP1LC3/LC3)-II in response to NCP BVDV infection was detected (Figure 1E).

Several studies have demonstrated that the induction of mitophagy upon virus infection can recruit adaptor proteins LC3-II and p62 to damaged mitochondria for the formation of mitophagosomes [18]. Here, immunofluorescence analysis showed a significantly increased translocation of LC3-II and p62 to damaged mitochondria in the NCP BVDV group at 48 hpi compared to that in mock-infected cells (Figures 1F, G). Together, these findings reveal that NCP BVDV infection induces the formation of mitophagosomes.

### Drp-1-mediated mitochondrial fission facilitates the progression of NCP BVDV infection

**Figure 2:**
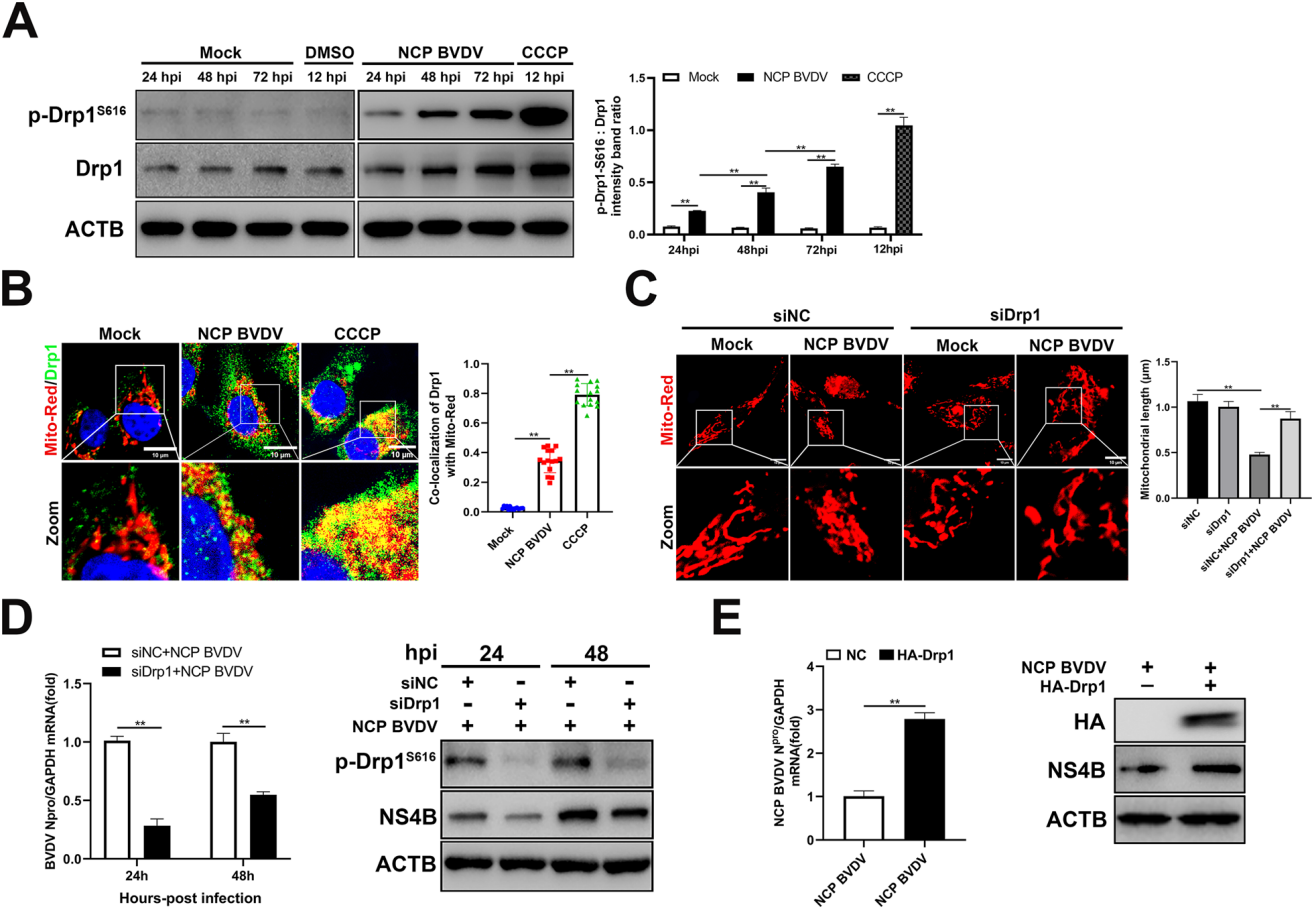
**Drp-1-mediated mitochondrial fission facilitates the progression of NCP BVDV infection.** **A** Western blot analysis of Drp1 and p-Drp1-S616 expression in mock and NCP BVDV-infected cells at indicated time point post infection. CCCP (10 μM) group was collected at 12 h post-incubation (hpi). **B** IFA analysis of the recruitment of Drp1 to damaged mitochondria in mock and NCP BVDV-infected (MOI = 5) cells at 48 hpi. (Scale bar = 10 μm). **C** MDBK cells were transfected with siDrp1 or siNC for 24 h and infected with NCP BVDV (MOI = 5), Mito Tracker Red-labeled mitochondrial morphology was observed by IFA assay. CCCP (10 μM) group was collected at 12 hpi. (Scale bar = 10 μm). The mean mitochondrial length was calculated using Image J software (Version 1.53t) (NIH). **D** MDBK cells were transfected with siDrp1 or siNC for 24 h. Then, the cells were infected with NCP BVDV (MOI = 5). qRT-PCR (left) and Western blot (right) assays were performed to determine the viral replication and progeny at 24 hpi and 48 hpi. **E** Viral replication levels in MDBK cells stably expressing Drp1 followed by NCP BVDV (MOI = 5) infection were detected by qRT-PCR (left) and Western blot (right) at 48 hpi. Data are given as means ± standard deviation (SD) from three independent experiments. *P* values were calculated using the Student *t* test. An asterisk indicates a comparison with the indicated control. *P < 0.05; **P < 0.01; ns: not significant.

Previous studies have shown that virus infection promotes upregulation of Drp1 and Drp1-S616 expression and translocation of Drp1 to damaged mitochondria prior to initiation of mitochondrial fission and mitophagy [16, 31]. To determine whether BVDV infection triggers Drp1-mediated mitochondrial fission, we detected the expression of Drp1 and Drp1-S616 in NCP BVDV-infected cells. Our data showed that NCP BVDV significantly stimulated upregulation of Drp1 and Drp1-S616 expression in a post infection time-dependent manner (Figure 2A). Next, we examined the translocation of Drp1 to mitochondria labelled with Mito Tracker in NCP BVDV-infected cells by IFA assay. As expected, NCP BVDV-infected cells displayed significantly enhanced mitochondrial translocation of Drp1, compared to mock-infected cells at 48 hpi (Figure 2B). Then, we depleted Drp1 via siRNA knockdown to investigate the role of Drp1 in BVDV-induced mitochondrial fission. Our data indicated that Drp-1-deficient MDBK cells upon NCP BVDV infection showed an elongated or enlarged mitochondrial morphology compared to NCP BVDV-infected cells alone (Figure 2C). Previous studies have shown that mitochondrial fission induced by viral infection is important for virus propagation [32, 33]. Thus, we speculate that the induction of mitochondrial fission is important for BVDV replication. To test this possibility, we examined BVDV replication in infected cells in the presence or absence of siDrp1 or siNC. qPCR and Western blot analysis indicated that knockdown of Drp-1 significantly suppressed NCP BVDV replication levels in cells (Figure 2D). In addition, qPCR and Western blot analysis indicated that Drp1 overexpression significantly stimulated mRNA levels of N^pro^ gene and NS4B protein levels (Figure 2E). Together, these results indicate that NCP BVDV infection induces Drp1-mediated mitochondrial fission to promote viral replication.

### Parkin-mediated mitophagy facilitates the progression of NCP BVDV infection

**Figure 3:**
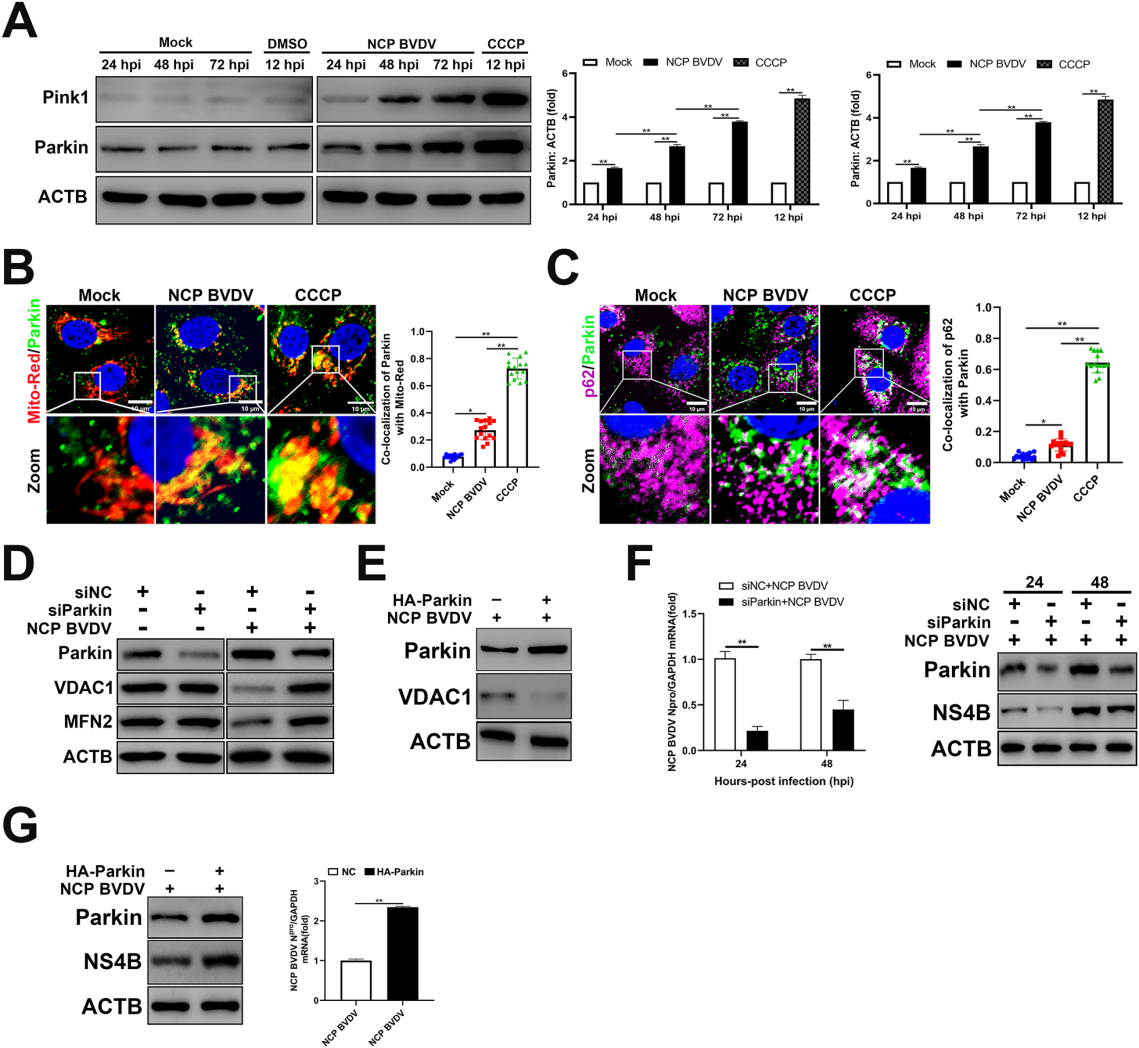
**Parkin-mediated mitophagy facilitates the progression of NCP BVDV infection.** **A** Western blot analysis of PINK1 and Parkin proteins in mock and NCP BVDV-infected cells (MOI = 5) at the indicated time points post infection. CCCP (10 μM) group was collected at 12 hpi. **B**, **C** IFA analysis of the recruitment of Parkin to damaged mitochondria (**B**) and co-localization of p62 with Parkin (**C**) in mock and NCP BVDV-infected (MOI = 5) cells at 48 hpi, as well as in CCCP treated cells (10 μM) at 12 hpi (Scale bar = 10 μm). **D** MDBK cells were transfected with siParkin or siNC for 24 h. Then, the cells were infected with NCP BVDV (MOI = 5), Western blot assays were performed to determine VDAC1 and MFN2 expression at 48 hpi. **E** Western blot analysis of VDAC1 expression in MDBK cells stably expressing Parkin following NCP BVDV infection at 48 hpi. **F** MDBK cells were transfected with siParkin or siNC for 24 h. Then, the cells were infected with NCP BVDV (MOI = 5), qRT-PCR (left) and Western blot (right) assays were performed to determine the viral replication and progeny at 24 hpi and 48 hpi. **G** MDBK cells stably expressing Parkin were infected with NCP BVDV (MOI = 5), Western blot (left) and qRT-PCR (right) assays were performed to determine the viral progeny and replication at 48 hpi. Data are given as means ± standard deviation (SD) from three independent experiments. *P* values were calculated using Student *t* test. An asterisk indicates a comparison with the indicated control. *P < 0.05; **P < 0.01; ns: not significant.

Previous studies have shown that PINK1 accumulates on MOM and selectively recruits Parkin to depolarized mitochondria when mitochondria lose the membrane potential [34]. Our data showed that NCP BVDV infection significantly stimulated the expression of PINK1 and Parkin in a post infection time-dependent manner (Figure 3A). Meanwhile, the colocalization of Parkin with fragmented mitochondria was observed in NCP BVDV-infected cells (Figure 3B).

Following translocation of Parkin to damaged mitochondria, recruitment of p62 is required for the clearance of mitochondria [35]. Here, recruitment of p62 to Parkin-containing mitochondria following NCP BVDV infection was observed (Figure 3C). Next, we investigated whether knockdown of Parkin can affect mitochondrial protein expression upon NCP BVDV infection. Our data showed that although NCP BVDV infection significantly decreased MFN2 and VDAC1 expression in siNC-transfected cells, knockdown of Parkin significantly abrogated the reduction of MFN2 and VDAC1 expression in NCP BVDV-infected cells (Figure 3D). Moreover, Parkin overexpression also significantly decreased VDAC1 expression compared to control cells (Figure 3E). Next, we investigated the role of mitophagy in BVDV replication. qPCR and Western blot analysis showed that knockdown of Parkin significantly reduced virus replication levels in NCP BVDV-infected cells at indicated time points compared to respective control cells (Figure 3F). In addition, Western blot and qPCR analysis indicated that Parkin overexpression significantly stimulated the expression of NS4B protein and mRNA levels of N^pro^ gene upon NCP BVDV infection (Figure 3G). Together, these data indicate that Parkin-mediated mitophagy facilitates NCP BVDV infection.

### NCP BVDV induces complete mitophagy

**Figure 4:**
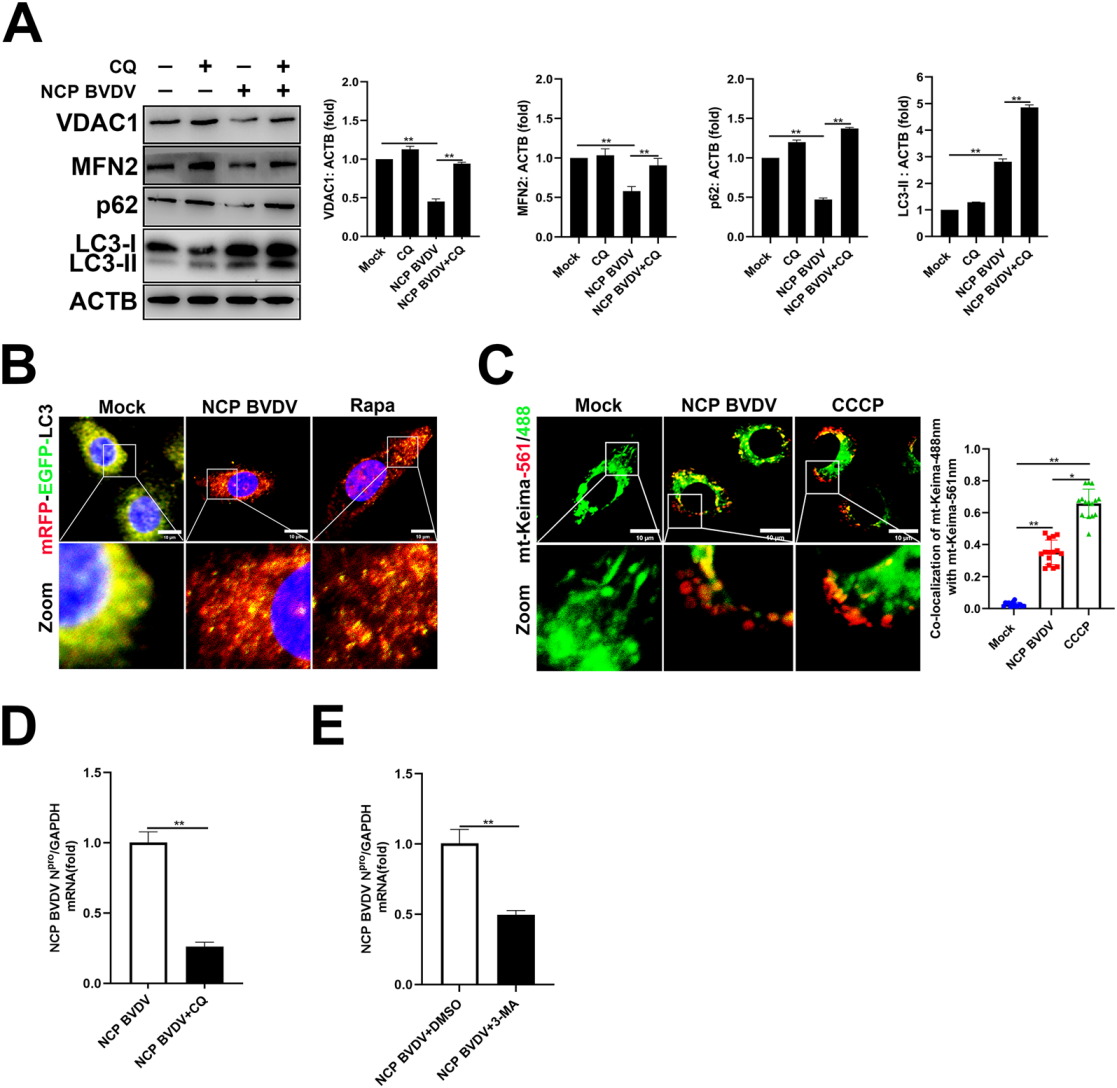
**NCP BVDV induces complete mitophagy.** **A** Western blot analysis of VDAC1, MFN2, p62 and LC3-II expression in NCP BVDV-infected (MOI = 5) cells in the presence or absence of CQ at 48 hpi. **B** IFA analysis of mRFP-EGFP-LC3 in NCP BVDV-infected (MOI = 5) cells or Rapamycin-treated (100 nM) cells at 48 hpi. (Scale bar = 10 μm). **C** IFA analysis of the recruitment of mt-Keima to mitochondria in NCP BVDV-infected (MOI = 5) cells at 48 hpi or CCCP-treated cells at 12 hpi. (Scale bar = 10 μm). **D** MDBK cells were infected with NCP BVDV (MOI = 5) in the presence or absence of CQ, and qRT-PCR assays were performed to determine the viral replication at 48 hpi. **E** MDBK cells were infected with NCP BVDV (MOI = 5) in the presence or absence of 3-MA, and qRT-PCR was performed to determine the viral replication at 48 hpi. Data are given as means ± standard deviation (SD) from three independent experiments. *P* values were calculated using the Student *t* test. An asterisk indicates a comparison with the indicated control. *P < 0.05; **P < 0.01; ns: not significant.

Previous studies have demonstrated that the fusion of mitophagosomes with lysosomes is the final step of mitophagy [17]. To monitor the autophagic flux upon BVDV infection, we performed p62 degradation assay to detect the accumulation of autophagosomes in the presence of chloroquine (CQ), a specific inhibitor of lysosomal degradation [36]. Our data showed that significantly enhanced expression of LC3-II in cells exposed to NCP BVDV was detected in the presence of CQ compared to untreated cells (Figure 4A). Importantly, CQ treatment reversed reduction of MFN2, VDAC1 and p62 in NCP BVDV-infected cells (Figure 4A). Recent studies have shown that tandem fluorescent-tagged LC3 (mRFP-EGFP-LC3) is a convenient assay for monitoring autophagic flux based on different pH stability of EGFP and mRFP fluorescent proteins [17, 37]. Here, we examined autophagic flux in NCP BVDV-infected cells by using the mRFP-EGFP-LC3 vector. Our results indicated that green puncta and yellow puncta significantly decreased in NCP BVDV-infected cells and Rapamycin-treated cells, indicating the elimination of autophagosomes due to complete autophagy. However, mock-treated cells showed more green puncta and yellow puncta (Figure 4B). To further monitor mitophagy flux, we expressed mt-Keima, a pH-sensitive fluorescent protein that presents green fluorescence under neutral environments such as normal mitochondria and red fluorescence under acidic pH when mitochondria are engulfed by lysosomes during mitophagy [38]. A far greater number of red dots appeared in NCP BVDV-infected and CCCP-treated cells than pre-transfected with mt-Keima, indicating that NCP BVDV infection induces complete mitophagy (Figure 4C). Next, we investigated the role of complete mitophagy in NCP BVDV replication. Our data showed that sharply decreased viral levels in NCP BVDV-infected cells were detected in the presence of CQ (Figure 4D) and 3-MA (Figure 4E), a specific inhibitor of the formation of autophagosomes [39]. These results clearly indicate that complete mitophagy is essential for NCP BVDV replication.

### E^RNS^ acts as a receptor of mitophagy to interact with LC3-II

**Figure 5:**
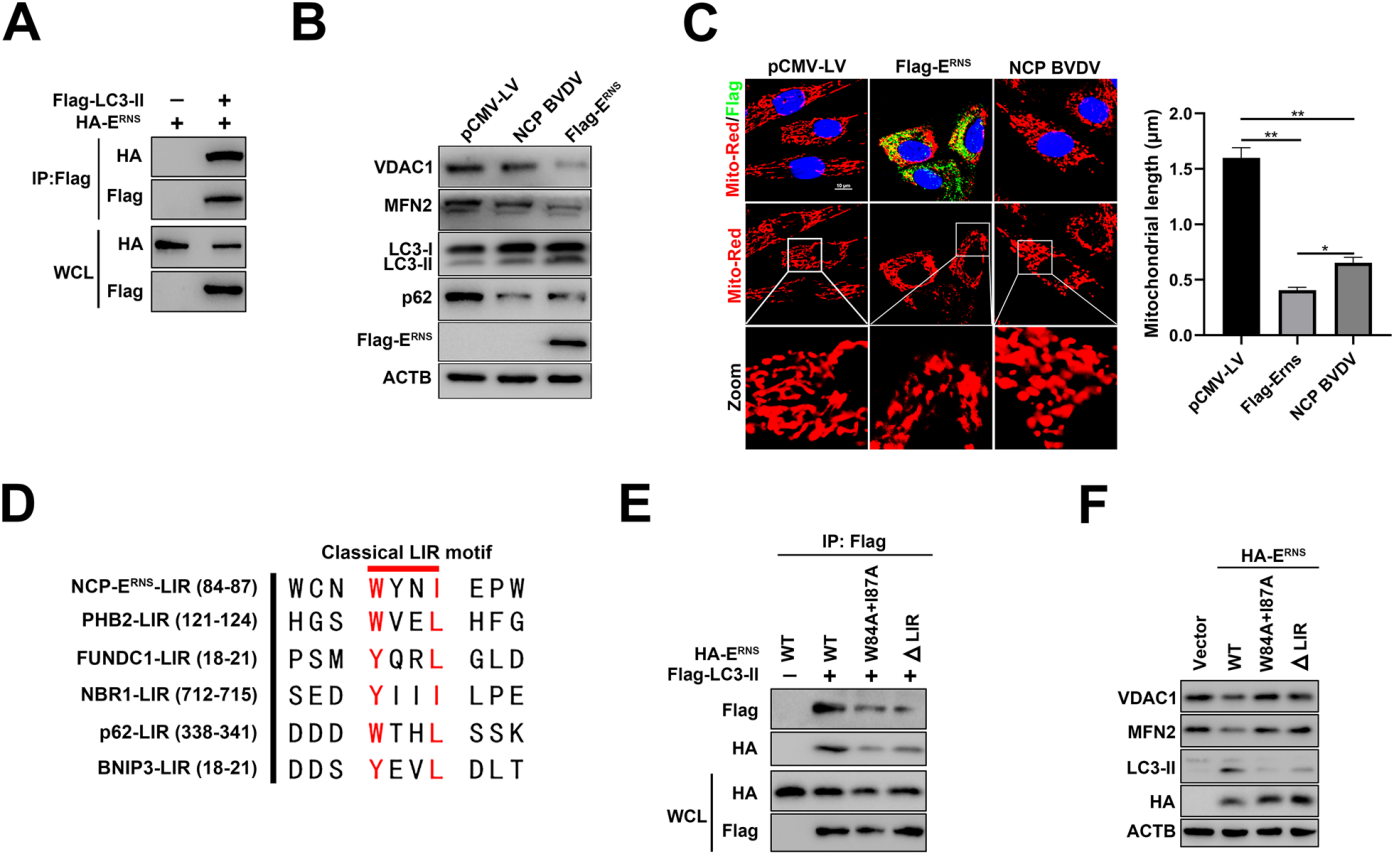
**E**^**rns**^** acts as a receptor of mitophagy to interact with LC3-II.** **A** HEK 293T cells were transfected with Flag-LC3-II and HA-E^RNS^. Cell lysates were immunoprecipitated with anti-Flag antibody and then analyzed by Western blot. **B** Western blot analysis was performed to detect VDAC1, MFN2, p62 and LC3-II expression in empty vector control group, NCP BVDV group and E^RNS^ overexpression group at 48 hpi. **C** IFA analysis of the recruitment of E^RNS^ to damaged mitochondria in MDBK cells stably expressing E^RNS^ at 48 hpi. NCP BVDV-infected (MOI = 5) cells were used as positive control. (Scale bar = 10 μm). **D** The sequences of the LIR motif (W/YxxI/L) in E^RNS^ were aligned manually with typical autophagy receptors. **E** HEK 293T cells were co-transfected with each HA-tagged WT E^RNS^ and its LIR mutants (E^RNS^ W84A+I87A and E^RNS^ΔLIR) with Flag-LC3-II. Cell lysates were immunoprecipitated with anti-Flag antibody and then analyzed by Western blot. **F** HEK 293T cells were transfected with HA-WT E^RNS^ and its LIR mutants. Cell lysates were harvested for Western blot analysis at 48 hpi. Data are given as means ± standard deviation (SD) from three independent experiments. *P* values were calculated using Student *t* test. An asterisk indicates a comparison with the indicated control. *P < 0.05; **P < 0.01; ns: not significant.

A previous study has shown that E^rns^ protein can colocalize with LC3-II, which suggests that E^rns^ may physically interact with LC3-II [4]. Similar results were obtained in this study where Flag-tagged-LC3-II did physically interact with HA-tagged-E^rns^ (Figure 5A). In addition, our data revealed that E^rns^ overexpression significantly decreased VDAC1, MFN2 and p62 expression, and increased LC3-II expression (Figure 5B), indicating that E^rns^ protein alone is sufficient to induce complete mitophagy. Furthermore, immunofluorescence analysis showed that E^rns^ overexpression and NCP BVDV infection both promoted mitochondrial fragmentation compared to the typical tubular mitochondria in mock-infected cells, respectively (Figure 5C). Previous studies have demonstrated that several proteins, including p62, FUNDC1, NBR1 and BNIP3L, containing a classical LIR (LC3-interacting region) motif with a conserved sequence of W/YxxL/I, are responsible for autophagy receptors interacting with LC3-II to regulate mitophagy [13]. In this study, we found that E^rns^ carried a typical conserved LIR motif W(84)xxI(87) (Figure 5D). To investigate whether the LIR motif alone can respond to E^rns^ interaction with LC3-II, a series of mutant forms of E^rns^ (E^rns^ W84A and I87A double-point mutant and E^rns^-ΔLIR mutant) were constructed. Our data demonstrated that mutations formed with E^rns^ resulted in impairment of binding of E^rns^ to LC3-II, indicating that E^rns^ LIR motif is involved in binding LC3-II (Figure 5E). Intriguingly, the LIR mutant-deficient E^rns^ failed to induce mitophagy (Figure 5F). Together, these results indicate that E^rns^ can serve as a receptor of LC3-II in mitophagy induction.

### NCP BVDV-induced mitophagy restricts MAVS-mediated type I interferon signaling

**Figure 6:**
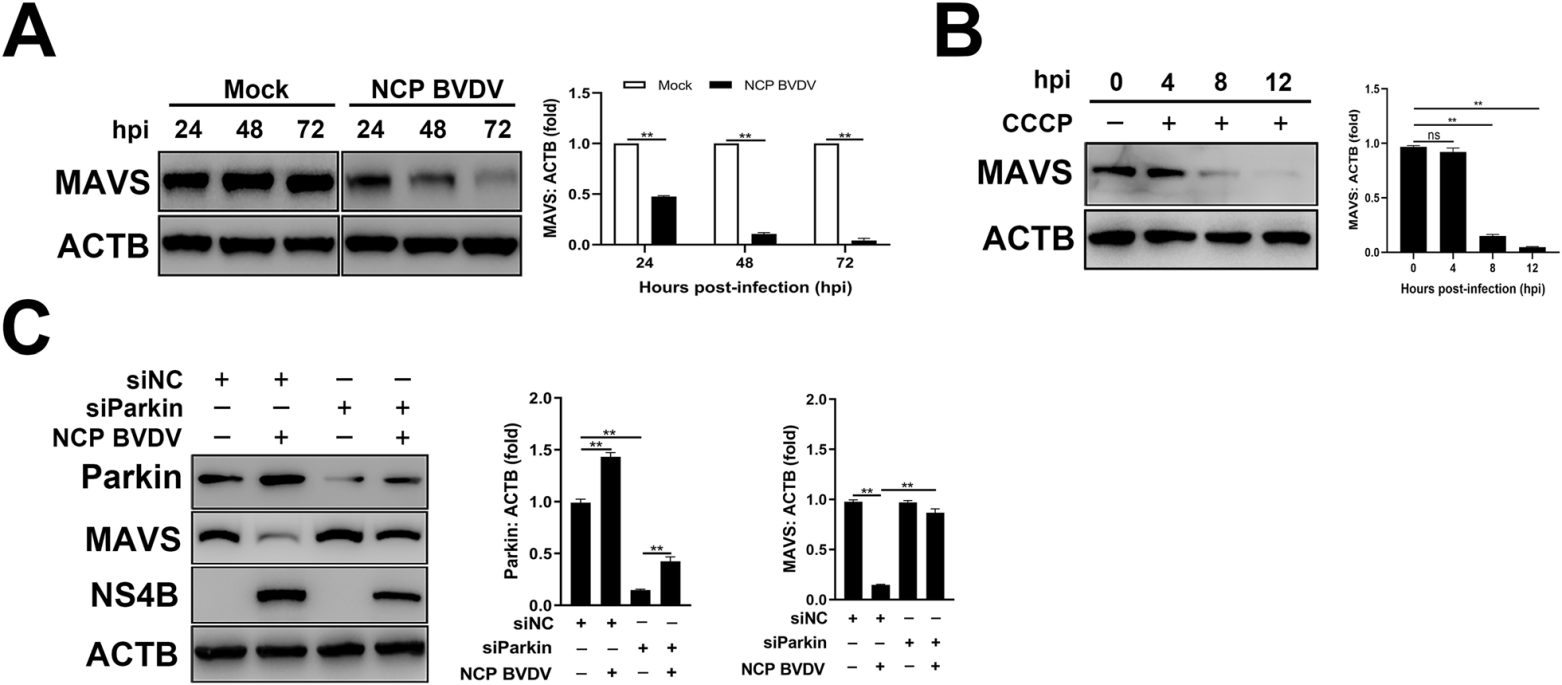
**NCP BVDV-induced mitophagy restricts MAVS-mediated type I interferon signaling.** **A** Western blot analysis of MAVS expression in NCP BVDV-infected (MOI = 5) cells at the indicated time points post-infection. **B** Western blot analysis of MAVS expression in CCCP-treated cells at 0 hpi, 4 hpi, 8 hpi and 12 hpi. **C** MDBK cells were transfected with siParkin or siNC for 24 h. Then, the cells were infected with NCP BVDV (MOI = 5), Western blot assays were performed to determine the MAVS, Parkin and NS4B expression at 48 hpi. Data are given as means ± standard deviation (SD) from three independent experiments. *P* values were calculated using the Student *t* test. An asterisk indicates a comparison with the indicated control. *P < 0.05; **P < 0.01; ns: not significant.

Mitochondrial antiviral signaling (MAVS) acts as a central signaling molecule in the RIG-I-like receptor (RLR) signaling pathway [40]. Ever-increasing amounts of evidence have demonstrated that the induction of mitophagy upon virus infection suppresses mitochondria-mediated antiviral innate immune response by degrading MAVS [40–42]. Here, Western blot assays were performed to examine endogenous MAVS levels in NCP BVDV-infected cells. Time-course analysis showed that endogenous MAVS levels were gradually downregulated in NCP BVDV-infected cells compared to mock-infected cells (Figure 6A). Similarly, CCCP-treated cells decreased MAVS expression in a time-dependent manner (Figure 6B). To determine the role of mitophagy induced by NCP BVDV infection in regulating MAVS expression, we detected MAVS expression in NCP BVDV-infected cells pre-transfected with siParkin or siNC. Our data showed that inhibition of mitophagy significantly abrogated the reduction of MAVS in NCP BVDV-infected cells. Importantly, inhibition of mitophagy also suppressed viral levels in NCP BVDV-infected cells (Figure 6C). Together, these results indicate that the induction of complete mitophagy upon NCP BVDV infection promotes MAVS degradation to restrict MAVS-mediated type I interferon signaling.

### NCP BVDV-induced mitophagy restricts cGAS-mediated type I interferon signaling

**Figure 7:**
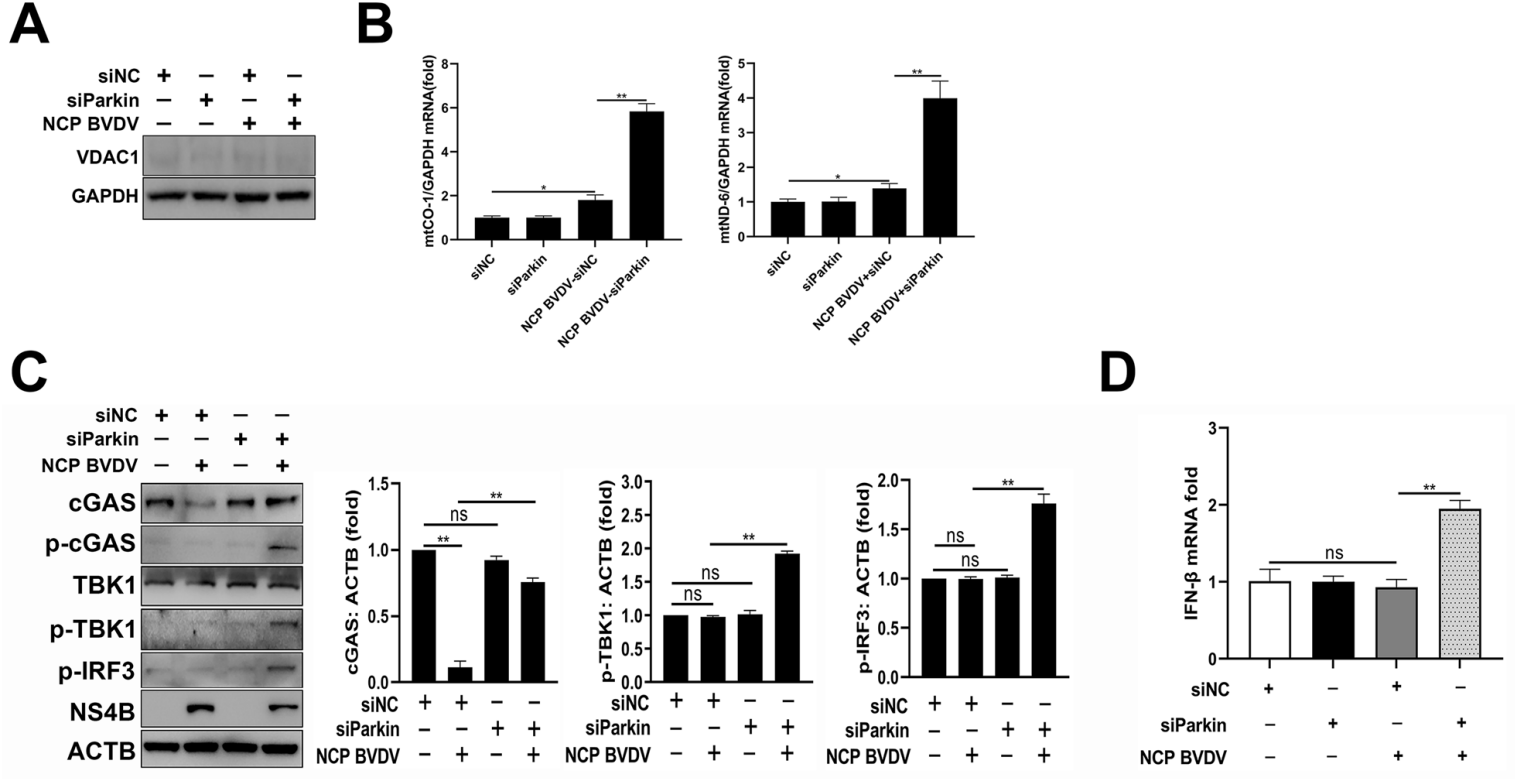
**NCP BVDV-induced mitophagy restricts cGAS-mediated type I interferon signaling.** **A** Western blot analysis of VDAC1 protein in cytoplasmic fractions from MDBK cells infected with NCP BVDV (MOI = 5) in the presence or absence of siParkin or siNC at 48 hpi. **B** MDBK cells were transfected with siParkin or siNC for 24 h. Then, the cells were infected with NCP BVDV (MOI = 5), qPCR assays were performed to determine the mtCO1 and mtND6 levels of mtDNA in cytoplasmic fractions isolated from the treated cells at 48 hpi. **C** MDBK cells were transfected with siParkin or siNC for 24 h. Then, the cells were infected with NCP BVDV (MOI = 5), Western blot assays were performed to determine the cGAS, p-cGAS, TBK1, p-TBK1, p-IRF3 and NS4B expression at 48 hpi. **D** MDBK cells were transfected with siParkin or siNC for 24 h. Then, the cells were infected with NCP BVDV (MOI = 5). RNA from each group was collected at 48 hpi, qRT-PCR assays were performed to determine the IFN-β expression in the indicated treatment cells. Data are given as means ± standard deviation (SD) from three independent experiments. *P* values were calculated using Student *t* test. An asterisk indicates a comparison with the indicated control. *P < 0.05; **P < 0.01; ns: not significant.

It has been demonstrated that damaged mitochondria can release mitochondrial DNA (mtDNA) into the cytosol to engage the DNA sensor cGAS, which promotes cGAS-STING-IRF3-dependent signaling to elevate ISG and type I interferon production [43]. Since NCP BVDV infection impaired mitochondria as observed in this study, we performed qPCR assays to determine the levels of cytosolic mtDNA following NCP BVDV infection. No detectable VDAC1 expression in purified cytoplasmic fractions from the cells confirmed there was no mitochondria contamination in cytoplasmic fractions (Figure 7A). In addition, our data revealed that NCP BVDV infection enhanced the levels of cytosolic mtDNA (*mt-CO1* and *mt-ND6*) compared to mock-infected cells (Figure 7B). However, knockdown of Parkin induced extensive cytosolic mtDNA levels in NCP BVDV-infected cells compared to untreated control cells (Figure 7B), suggesting that mitophagy can prevent further damage of mitochondria and release of mtDNA from mitochondria to the cytosol. Moreover, our data showed that no significant changes of p-cGAS, p-TBK1 and p-IRF3 (Figure 7C) as well as interferon-β (IFN-β) expression (Figure 7D) were detected in NCP BVDV-infected cells, which was consistent with the disability of induction of IFN-β upon NCP BVDV infection [3]. However, inhibition of mitophagy significantly enhanced the activation of cGAS-TBK1 signaling (Figure 7C) and IFN-β expression (Figure 7D) in NCP BVDV-infected cells. Collectively, these data suggest that the induction of complete mitophagy by NCP BVDV can eliminate the cytosolic mtDNA and promote cGAS degradation to restrict cGAS-TBK1-mediated type I interferon.

### NCP BVDV-induced mitophagy restricts ROS-mediated inflammatory responses

**Figure 8:**
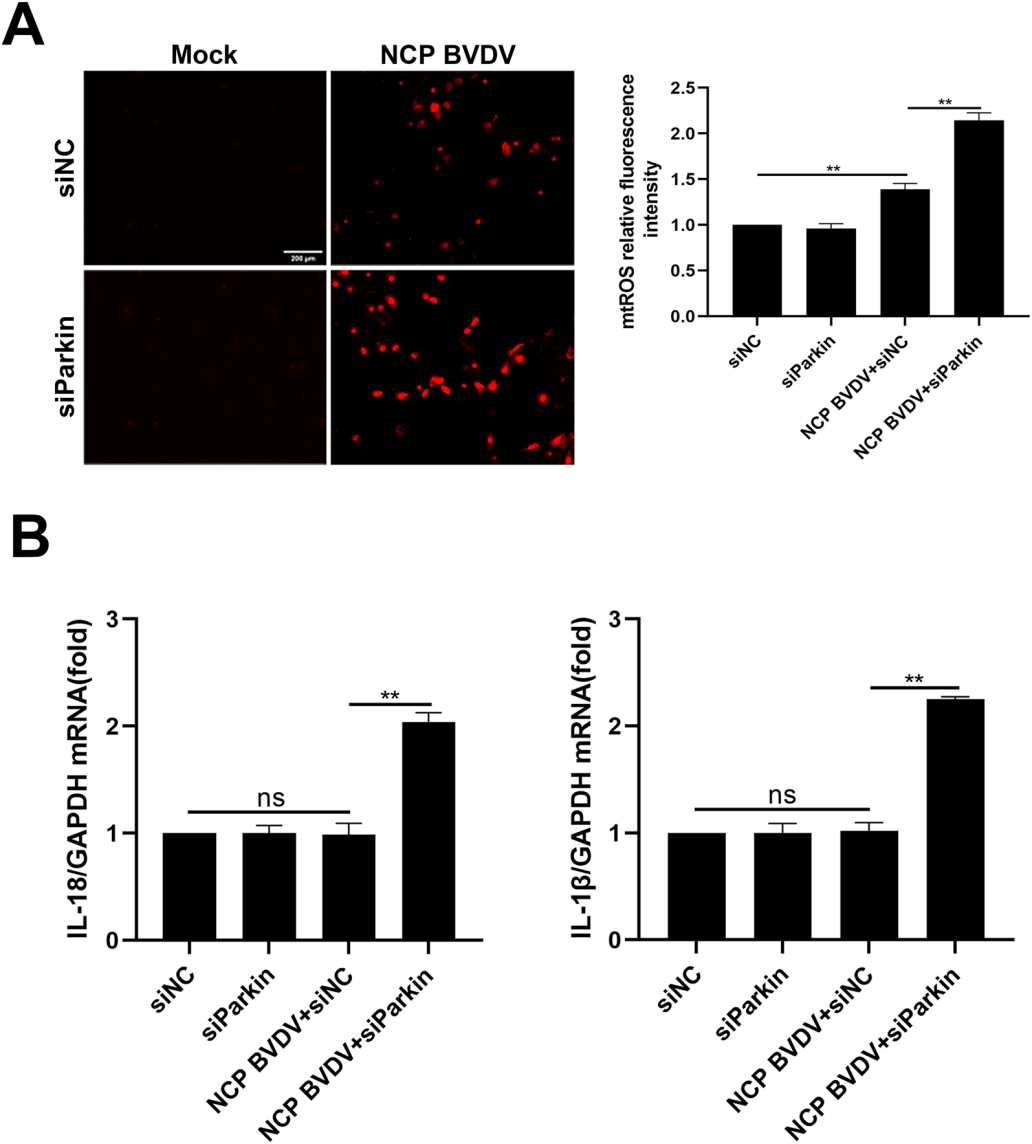
**NCP BVDV-induced mitophagy restricts ROS-mediated inflammatory responses.** A MDBK cells were transfected with siParkin or siNC for 24 h. Then, the cells were infected with NCP BVDV (MOI = 5). IFA analysis was performed to determine the mtROS levels in mock- and NCP BVDV-infected (MOI = 5) cells at 48 hpi. (Scale bar = 200 μm). The mean fluorescence intensity of mtROS was measured using the Image J software. **B** MDBK cells were transfected with siParkin or siNC for 24 h. Then, the cells were infected with NCP BVDV (MOI = 5). RNA from mock- and NCP BVDV-infected (MOI = 5) cells was collected at 48 hpi, then qRT-PCR assays were performed to determine the IL-1β and IL-18 expression in indicated treatment cells. Data are given as means ± standard deviation (SD) from three independent experiments. *P* values were calculated using Student *t* test. An asterisk indicates a comparison with the indicated control. *P < 0.05; **P < 0.01; ns: not significant.

Impaired mitochondrial structural and functional integrity is accompanied with excessive mitochondrial reactive oxygen species (mtROS) production and inflammasome-activating signals [44]. Here, we examined the relationship between mtROS and NCP BVDV-induced mitophagy. Immunofluorescence analysis showed that NCP BVDV infection resulted in mtROS accumulation compared to the mock infection group, while inhibition of mitophagy enhanced the mtROS accumulation compared to NCP BVDV-infected cells alone (Figure 8A). Importantly, qRT-PCR analysis showed that, although there were no significant changes for IL-1β and IL-18 mRNA expression in NCP BVDV-infected cells compared to mock-infected cells, inhibition of mitophagy enhanced these cytokines expression in NCP BVDV-infected cells (Figure 8B). Collectively, these data suggest that the induction of mitophagy may contribute to restricting the ROS-mediated inflammatory responses.

### Role of NCP BVDV-induced mitophagy on apoptosis

**Figure 9:**
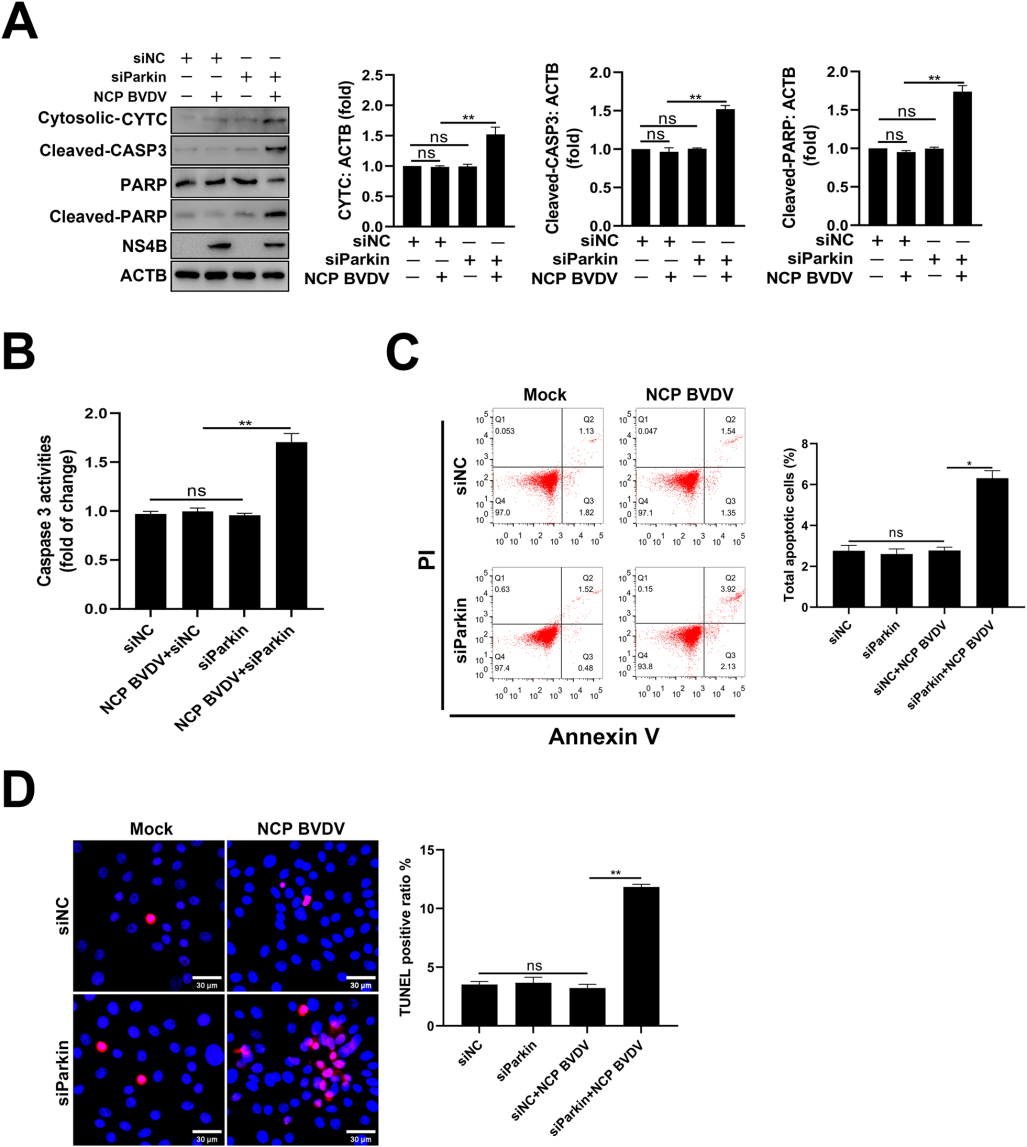
**Role of NCP BVDV-induced mitophagy on apoptosis.** **A** MDBK cells were transfected with siParkin or siNC for 24 h. Then, the cells were infected with NCP BVDV (MOI = 5), Western blot assays were performed to determine the CYTC, cleaved-caspase3, cleaved-PARP and NS4B expression at 48 hpi. **B** MDBK cells were transfected with siParkin or siNC for 24 h. Then, the cells were infected with NCP BVDV (MOI = 5), the activities of caspase-3 were determined at 48 hpi. **C**, **D** MDBK cells were transfected with siParkin or siNC for 24 h. Then, the cells were infected with NCP BVDV (MOI = 5), apoptosis was detected by flow cytometry (**C**) and TUNEL (**D**) assays at 48 hpi, respectively. Data are given as means ± standard deviation (SD) from three independent experiments. *P* values were calculated using the Student *t* test. An asterisk indicates a comparison with the indicated control. *P < 0.05; **P < 0.01; ns: not significant.

Previous studies have shown that mitochondrial dynamics is integrally linked to apoptosis [45]. Our data showed that NCP BVDV infection had no significant effects on the release of cytochrome C (CYTC) from mitochondria to the cytosol, cleavage of caspase-3 and poly (ADP-Ribose) polymerase (PARP) proteins (Figure 9A), as well as the activities of caspase-3 (Figure 9B). Intriguingly, inhibition of mitophagy induced massive cytosolic cytochrome C release from mitochondria and promoted the cleavage of caspase-3 and PARP proteins (Figure 9A), as well as the activities of caspase-3 (Figure 9B) in NCP BVDV-infected cells. Correspondingly, NCP BVDV infection failed to induce apoptosis as seen by flow cytometry (Figure 9C) and TUNEL assays (Figure 9D). However, knockdown of Parkin promoted apoptosis in NCP BVDV-infected cells (Figure 9C, D). Collectively, these results demonstrate that NCP BVDV blocks the induction of apoptosis via mitophagy.

## Discussion

Mitophagy is an important mitochondrial quality control mechanism to eliminate damaged mitochondria [46]. Mitochondrial dynamics and mitophagy are two critical arms, which are required to maintain mitochondrial homeostasis [47]. Previous studies have shown that some viruses can induce mitophagy to evade the innate immune response for their persistent infection [33, 48, 49]. Here, we demonstrate that NCP BVDV stimulates upregulation of Drp1 and Drp1 (Ser616) expression, and promotes the recruitment of Drp1 to damaged mitochondria, which lead to mitochondrial fission and subsequent clearance of damaged mitochondria by Parkin-dependent complete mitophagy.

Given the findings that some viral proteins can translocate to mitochondria during viral infection [13, 50, 51], it is reasonable to speculate that apart from utilizing host mitophagic machinery to trigger mitophagy, some viruses can directly induce mitophagy via their own viral factors. In this study, based on the amino acid sequence analysis between autophagy receptor and E^rns^, we found that E^rns^ is necessary and sufficient to induce mitophagy as like viral particles. However, this finding does not exclude the possibility that other viral proteins may also facilitate the induction of mitophagy.

It has been demonstrated that the failure to stimulate an efficient innate immune response is the primary cause for persistent infection during NCP BVDV infection [52]. Many viruses have developed several strategies to disable MAVS signaling and interrupt RLR signaling to evade innate immune responses [13, 53]. Although the ability of NCP BVDV to suppress interferon responses may be a key determinant for persistent infection in the host, the interaction between NCP BVDV and innate immune responses remains largely unknown. In this study, we demonstrate that NCP BVDV infection significantly inhibits the expression of MAVS compared to that in mock-infected cells. Inhibition of mitophagy significantly abrogates NCP BVDV-induced reduction of MAVS. Overall, we demonstrate that NCP BVDV-induced mitophagy may play an important role in the inhibition of MAVS-mediated innate immune response.

Previous studies have indicated that damaged mitochondria can release mtDNA into the cytosol, which can be sensed by cGAS-STING pathway to induce innate immunity [54]. There are several possible mechanisms by which mtDNA leaks into the cytosol to induce cGAS-STING signaling-mediated IFN responses [55, 56]. Here, we demonstrate that although NCP BVDV induces the release of mtDNA and decreases cGAS expression compared to that in mock-treated cells, inhibition of mitophagy further increases the cytosolic mtDNA levels in NCP BVDV-infected cells, which is concomitant with strong cGAS and p-cGAS signaling activation and IFN-β expression. These findings are in agreement with earlier reports wherein inhibition of initiation of mitophagy results in accumulation of damaged mitochondria, which eventually promotes more mtDNA release into the cytosol [29, 57]. Importantly, our results reveal that induction of mitophagy suppresses the activation of cGAS signaling during NCP BVDV infection.

It has been demonstrated that damaged mitochondria generate excessive mtROS, which promotes the activation of inflammatory responses [58, 59]. Clearance of impaired mitochondria via mitophagy can inhibit inflammation responses [60]. Here, our data reveal that NCP BVDV infection has no significant effects on expression of IL-1β and IL-18. These results are consistent with previous studies, which demonstrate that NCP BVDV is associated with no inflammatory responses [61–64]. Given the findings that mitophagy acts as a brake on inflammasome signaling by removing dysfunctional mitochondria, and that the attenuated inflammatory responses promote viral evasion of the host antiviral innate immunity [65], it is reasonable to deduce that the expression levels of inflammatory cytokines in response to NCP BVDV infection may be closely associated with induction of mitophagy.

Apoptosis can confer advantages to virus-infected hosts by preventing cell-to-cell spread, which results in the death of infected cells and inhibits viral infection to continue [46, 66]. It has been demonstrated that NCP BVDV prevents initiation of the apoptotic cascade via the intrinsic pathway which involves the mitochondria [5, 67]. Although the molecular mechanisms underlying pathogenesis of NCP BVDV are not yet fully understood, it is inferred that NCP BVDV have evolved strategies to block the natural function of the apoptotic pathway. Previous studies have indicated that damaged mitochondria can release cytochrome C and trigger cytochrome C-mediated apoptosis [68]. Hence, a rapid turnover and clearance of damaged mitochondria are needed to confound imminent cell death due to aggravated mitochondrial injury during viral infection. In agreement with this assumption, we observe a surge in mitochondrial-apoptotic signaling and resultant death of NCP BVDV-infected cells upon inhibition of mitophagy. Further studies are required to determine if abrogation of Parkin-mediated mitophagy promotes specific death of NCP BVDV-infected cells and alleviates persistent NCP BVDV infection in vivo. Moreover, other intracellular mechanisms in addition to mitophagy underlying distinct modulating apoptosis in response to NCP BVDV infection may be possible and must be further studied.

**Figure 10:**
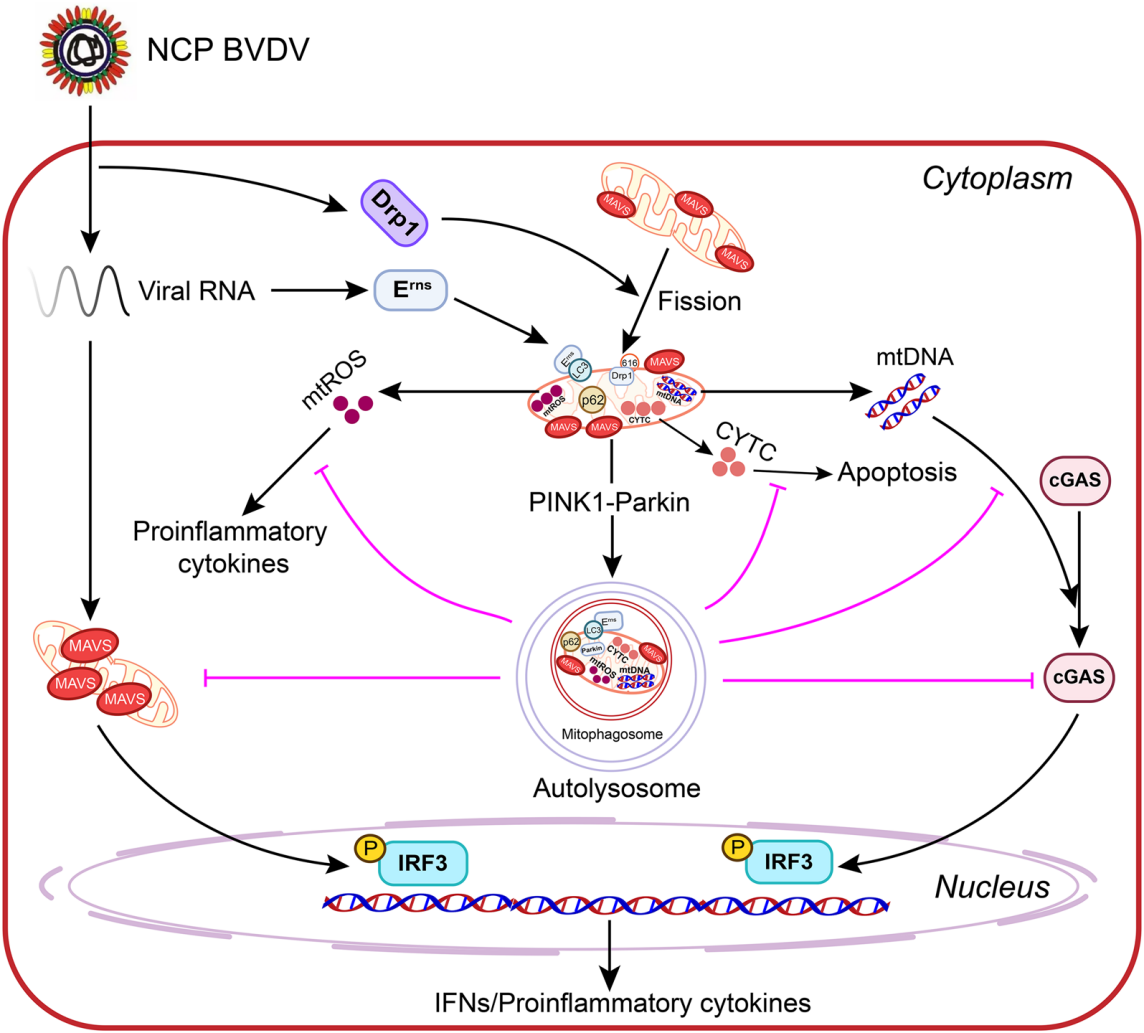
**Model of the induction of mitophagy by NCP BVDV to suppress the antiviral innate immune response and apoptosis.** NCP BVDV infection promotes the translocation of Drp1 to damaged mitochondria and the phosphorylation of Drp1 at the 616 site to mediate mitochondrial fission for viral replication. Subsequently, the Parkin-mediated complete mitophagy participates in clearance of damaged mitochondria to facilitate viral replication. In addition, the release of mitochondrial contents including mtDNA, mtROS and CYTC from damaged mitochondria can be effectively eliminated by complete mitophagy to inhibit type I interferon signaling, inflammatory responses and apoptosis, which lead to viral evasion of the host antiviral innate immunity.

Collectively, our results establish a novel link between mitochondrial dysfunction, mitophagy, innate immune responses and apoptosis in response to NCP BVDV infection (Figure 10), thereby suggesting that interventions aimed at blocking mitochondrial dynamics and/or decreasing the clearance of damaged mitochondria can provide promising therapeutic targets for abrogating NCP BVDV-mediated persistent infection.

## Data Availability

The datasets analyzed in this study are available from the corresponding authors upon reasonable request.
